# The Influence of Mobility Training on the Myofascial Structures of the Back and Extremities

**DOI:** 10.3390/jcm13020329

**Published:** 2024-01-06

**Authors:** Gunda Slomka, Robert Schleip, Peter Frei, Nicolas Kurpiers, Michael Krämer, Katharina Bauermeister, Wolfgang Bauermeister

**Affiliations:** 1 Institute of Sport Science, University of Hildesheim, Universitätsplatz 1, 31141 Hildesheim, Germany; gundaslomka@gmx.de (G.S.); peter.frei@uni-hildesheim.de (P.F.); kurpiers@uni-hildesheim.de (N.K.); 2 Conservative and Rehabilitative Orthopedics, Department Sport and Health Sciences, Technical University of Munich, Campus D, Georg-Bauchle-Ring 60/62, 80992 München, Germany; katharina.bauermeister@tum.de; 3 Department of Medical Professions, Diploma Hochschule, 37242 Bad Sooden-Allen, Germany; 4 Institute for Materials Technology, Technical University Darmstadt, Grafenstrasse 2, 64283 Darmstadt, Germany; michael.kraemer@tu-darmstadt.de; 5 Department of Sports, Physical and Rehabilitative Medicine, Kharkiv National Medical University, 4 Nauky Avenue, 61022 Kharkiv, Ukraine; v.bauermayster@knmu.edu.ua

**Keywords:** fascia research, connective tissue, ultrasound elastography, range of motion (ROM), mobility training, prevention, load compensation, well-being

## Abstract

Background: The subject of the study was the effect of a multicomponent program (Mobility Routine) on muscular and fascial stiffness, flexibility, subjective well-being, and body perception. Methods: The assumption was that high physical stress affects myofascial structures and joint range of motion. The assessment of myofascial stiffness employed a Shear Wave Elastography. The joint flexibility, pressure pain threshold, and subjective experiences with regard to tension, pain, and general discomfort were documented. Results: In the CT group, a greater increase in stiffness was measured in fewer measurement areas compared to the MR group. MR demonstrated superior gains in flexibility compared to CT. Both groups experienced significant reductions in pain, tension, and discomfort. In conclusion, repetitive motion patterns akin to CT lead to increased myofascial stiffness, whereas MR yields more balanced stiffness development, compensates for asymmetries, and improves body awareness. Conclusions: Hence, this study highlights the advantages of mobility training over Crosstrainer exercises and provides valuable insights for the recommendation of training regimens aiming at the enhancement of musculoskeletal functionality and overall well-being.

## 1. Introduction

For over a decade, there has been an intensified research focus on the fascial system. While it was previously assumed that this tissue, described as a passive structure, could not be influenced through training, contemporary knowledge asserts the trainability of fasciae. They adapt to daily life and training stimuli, and, with specific training stimuli, we are capable of molding the fascial system and consciously altering its mechanical and metabolic properties. A systematic review conducted by Wilke [1] documented that a substantial proportion of sports-specific injuries pertains to injuries of the fascial system (86.4% myotendinous lesions; 31.1% myofascial lesions; and 12.7% isolated muscular lesions). The present literature has documented the potential of exercise programs encompassing strength and stretching routines to effectively prevent injuries [2,3]. Accordingly, the Association of Substitute Health Funds in Germany (VDEK) actively supports preventive exercise initiatives and certification bodies evaluate and endorse relevant course systems accordance with §20 of the Prevention Act. However, few concepts have evidence-based proof regarding their preventive merits [4]. The Santa Monica Sports Medicine Foundation (SMSMF) and the Oslo Sports Trauma and Research Centre (OSTRC) developed a complete warm-up program in 2006 to prevent injuries in amateur soccer players. The study results show that the FIFA 11+ warm-up significantly prevents soccer injuries [5,6,7]. However, the general study situation on the effect of exercise programs as injury prevention is heterogeneous. Van Mechelen [8] could not present significant results of a warm-up program in terms of injury prevention in a study with runners, whereas a review by Woods et al. [9] describes the positive preventive effect of a warm-up routine directly before training. James et al. [10] highlighted an anti-inflammatory effect of exercise in the autochthonous back muscles of animals (mice). Presently, there is no proof of the effect of a complex exercise program on changes in tissue stiffness or joint range of motion or compensation for imbalances. In health, fitness, competitive sports, or even in therapy, exercise programs like Intervention Measure I (Mobility Routine/MR) are common and widely used. Despite this great popularity, scientific data are rare. Based on the anatomical and physiological results of fascial research, basic techniques for movement have been developed on which future research could build.

The research methods were used to investigate the following questions: Does a mobility training intervention change tissue stiffness, joint range of motion, and compensate for possible imbalances? Do the interventions influence the pressure pain threshold and the overall well-being?

## 2. Materials and Methods

### 2.1. Participants

A total of 73 individuals were initially enrolled in this research endeavor. We built an experimental cohort comprising individuals subjected to considerable physical demands, specifically officers affiliated with the State Criminal Police Office and the Special Deployment Command (SEK) in Lower Saxony, Germany. Given their substantial weekly physical exertion (averaging 24.7 h), these participants represent a clientele exposed to intense and potentially excessive physical stress. Five subjects were excluded from the study due to illness. All participants provided an updated medical fitness certificate (Erl. MiP 25.4-12506 v.20.12.2013, in the most current iteration). All participants signed an informed consent form prior to the study. All participants were simultaneously enrolled in the study. Data collection occurred over a multi-day testing period (5 days each). The intervention period for all participants spanned 12 weeks, from late May to early September.

Inclusion Criteria:

Police officer in the Special Deployment Command (SEK).

Age > 21 years.

Successful completion of the medical SEK fitness examination (Erl.MiP 25.4-12506 v.20.12.2013 in the current version).

Exclusion Criteria:

Intervention interruption due to illness for >14 days.

The interventions (MR and CT training) were scheduled for 12 weeks: Week 22 to Week 35 (2019).

T1 (Pre-test Period): 27 May to 1 June 2019.

T2 (Post-test Period): 26 August to 30 August 2019.

A total of 73 SEK officers were included: n = 73.

Intervention Group 1—MR Training: n = 52.

Intervention Group 2—CT Training: n = 21.

Five participants were excluded from the MR group during the intervention period (1× vasectomy, 1× general pain, 1× vacation during T2, 1× irregular training, and 1× knee bursitis).

The relevant sample size for the study was:

Entire cohort: n = 68.

Intervention Group 1—MR Training: n = 47.

Intervention Group 2—CT Training: n = 21.

Cohort from the State Criminal Police Office/Special Deployment Command (SEK) (Table 1).

The study was conducted according to the guidelines of the Declaration of Helsinki and approved by the Ethics Committee of the University of Hildesheim, Department 1, under reference number 138 (received on 28 April 2020). The study protocol was registered with the German Clinical Trials Register (DRKS00032581).

**Figure 1:**
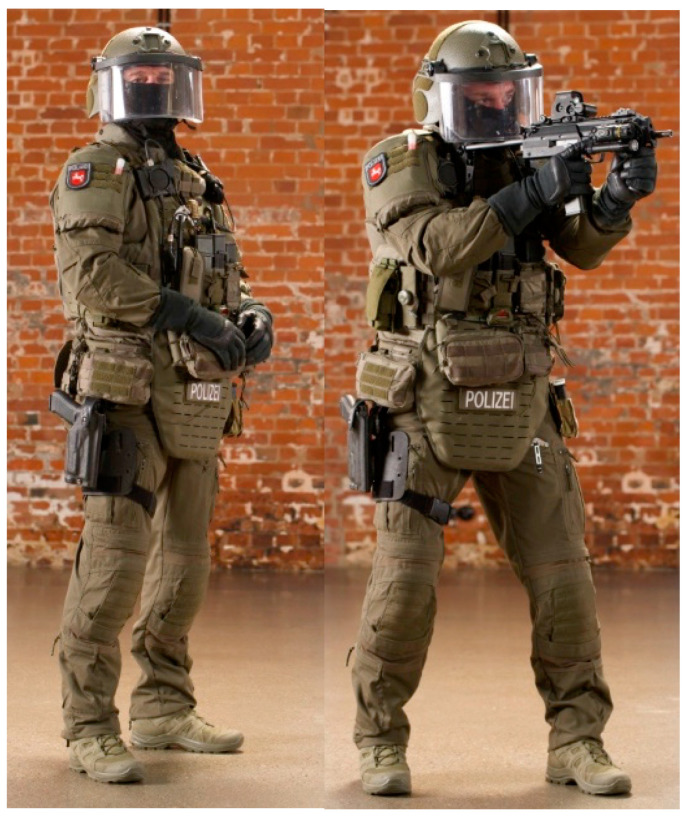
SEK officer with protective clothing.

This intervention study was designed with a gender-homogeneous cohort comprising exclusively male participants, aiming to mitigate potential confounding effects associated with cycle-related fluctuations in sex hormones on myofascial stiffness. The primary focus of the investigation was to assess the impact of mobility training on myofascial structures. Consequently, Intervention Group 1, undergoing a mobility routine, was maximized in size (n = 47). In contrast, the comparison group, undergoing cross-trainer training, was deliberately minimized (n = 21) to facilitate statistically robust conclusions. The constraints of the selected cohort (Specialized Police Unit) limited the feasibility of achieving an overall larger sample size (Table 2 and Figure 1).

### 2.2. Intervention

The test group was divided into the Mobility Routine (MR/n = 47) and Crosstrainer (CT/n = 21) groups. Over a 12-week period, both groups exercised three times per week for 30 min. The MR group performed video file guided mobility training based on six basic techniques. These basic techniques were developed based on anatomical, physiological, and pathophysiological assumptions regarding mechanisms of action as a working hypothesis. These techniques consist of exercises that aim to increase range of motion (1), to generate pressure variations (2), stretching exercises (3), to aid orientation to the myofascial lines (4), to increase body heat (5), and to integrate rotational and shear motion techniques (6). Together, these six techniques form the basic framework of a 35 min MR workout. The control group (CT group) completed a conventional Crosstrainer (CT) workout. The crosstrainer intervention, serving as an active control, aimed to assess the impact of general cardiovascular exercise, incorporating basic techniques such as an increase in body heat (5) and rotational and shear motion techniques. While the lower extremities executed a cyclic step-up motion, participants maintained hand contact with a secured handrail, facilitating a cross-pattern movement of both the arms and legs. This intervention spanned a twelve-week duration, with participants engaging in elliptical trainer sessions three times per week, each lasting 30 min. Importantly, the crosstrainer intervention served as a reference group, allowing the evaluation of specific effects of mobility training on myofascial structures compared to a general cyclic cardiovascular activity. The crosstrainer group adhered to instructions to maintain contact with the handrail, and the training intensity was set at a moderate level, specifically targeting basic endurance. This intensity was determined based on individual heart rate data for basic endurance training, which subjects had previously collected for their endurance training. It is noteworthy that the exercise program for the comparison group exclusively emphasized the basic techniques of warmth (5) and rotation and shear motion techniques (6).

**Figure 2:**
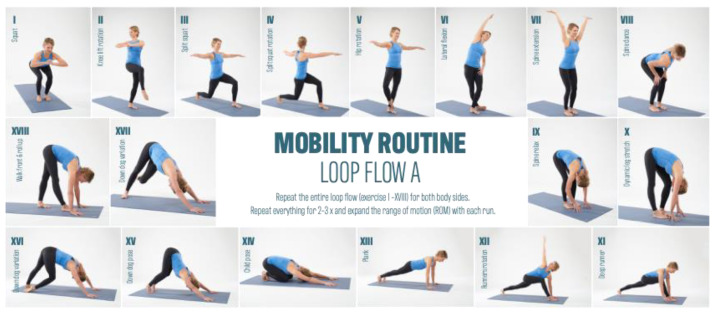
Mobility Routine Flow A.

**Figure 3:**
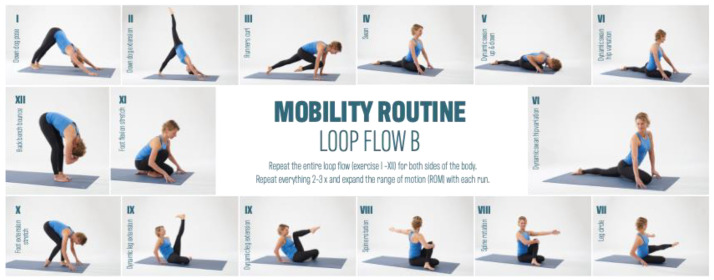
Mobility Routine Flow B.

**Figure 4:**
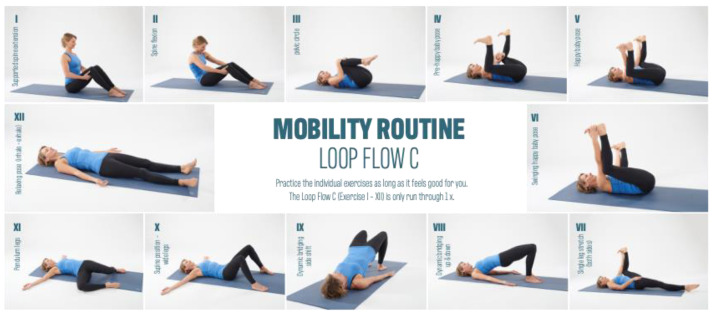
Mobility Routine Flow C.

Both groups trained with at least one day of intervention break between training sessions. The main interest of the study was to investigate the effect of MR training on the myofascial structures of the body. Figure 2, Figure 3 and Figure 4 provides an overview of the MR training.

**Figure 5:**
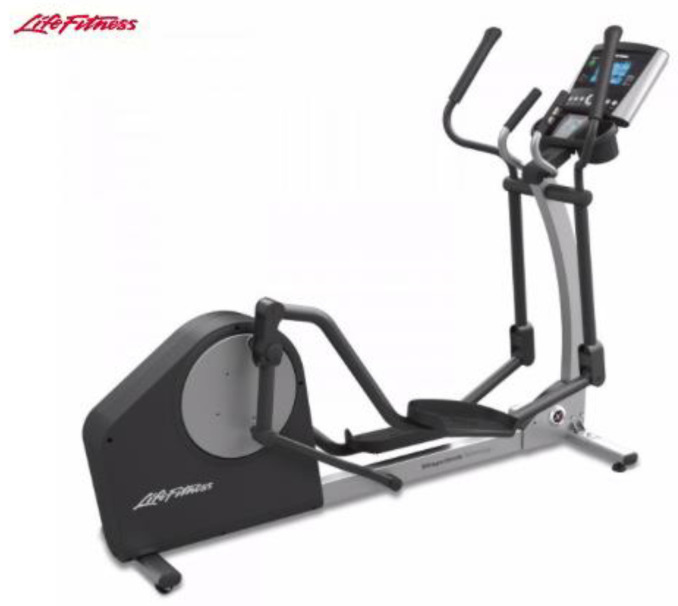
Crosstrainer—Intervention device Group II. Note: For the 12-week intervention period, three Lifefitness crosstrainers (Elipsen crosstrainer of the company Lifefitness—series activate) were made available at the office.

Figure 5 shows a cross-trainer. The training device of the second intervention group. Other Crosstrainers from other manufacturers at other locations were allowed to be used. Both the provided crosstrainer from Lifefitness and the devices used by other manufacturers are based on a foot–leg movement in elliptical paths that minimize joint impact compared to running. It is a full-body workout that trains both the legs, buttocks, and arms in over-cross movements. The individual intensity can be controlled via a resistance regulator.

All individual exercises are strung together to create an exercise flow. This flow is run through and routinized several times in the loop system before a new loop flow begins.

The entire Mobility Routine and background on the basic techniques can be read in the monograph accompanying this article (link to the monograph at the end of this article, under: Data Availability).

### 2.3. Measurement Technique

Shear Wave Elastography (SWE) was the chosen method to assess shifts in tissue stiffness. Collecting quantifiable data for myofascial stiffness using SWE is a novelty in sports science and sports therapy work [11,12,13]. To date, the method has been predominantly used in internal medicine and tumor diagnostics [14,15]. It allows assessment of the stiffness of the myofascia at several centimeters’ depth. The probe of the Resona 7 Ultrasound System (Mindray Bio-Medical Electronics Co., China) was positioned at the respective areas during examination, characterized by minimal tissue pressure and zero movement [16]. A pivotal metric in this analysis is the Motion Stability Index (M-STB), which gauges the extent of motion artifacts. The quality of measurements is described on a scale from 0 (indicating poor quality) to 5 (denoting optimal quality). For the purpose of measurements, exclusively cine recordings with a top-tier five-star rating were considered. Facilitating an expansive elastogram spanning several cm^2^ (approximately 3.3 cm × 2.5 cm/large area), this device provides comprehensive insights. Young’s Elasticity Modulus (E) serves as a metric to assess tissue stiffness, encompassing the entirety of the area of interest (indicated in kPa). The examination comprised twenty measurement regions spanning the trunk and extremities. Moreover, for the thoracolumbar fascia (TLF), the analysis was segregated into muscle and fascia components. Table 3 shows a total of 22 measurement regions.

The values of the different stiffnesses are color-coded in the elastogram (Figure 4). Warm colors indicate measurement areas with low elasticity (high stiffness), whereas the cold colors represent soft tissue.

The examinations were carried out by the same experienced examiner for all measurements. Parker ultrasound gel was used. To avoid movement artifacts, a special holding arm was used to place the probe. Quality assurance: The measurements were performed using ample amounts of ultrasound gel, applying minimal pressure, waiting for a steady image with almost identical E-values.

SWE is operator independent, whereas strain elastography is an operator-dependent measure. Wu and colleagues [17] reported excellent interrater reliability for RF (ICC 0.987), Vastusmedialis muscle (VM) (ICC 0.963), Vastus lateralis muscle (VL) (ICC 0.952), BF (ICC 0.981), GM (ICC0.953), and Lateral head of gastrocnemius muscle (GL) (ICC 0.968). Shear wave properties of VM and GM, assessed by Dubois et al. [11] at rest and during passive stretching, were reliable and, especially at rest, well reproducible (interrater reliability ICC = 0.91–0.87; intrarater reliability ICC = 0.94–0.91). For the RF, the intra-rater reliability (ICC = 0.93–0.94), inter-day reliability (ICC = 0.81–0.91), and inter- rater reliability (ICC = 0.95) were excellent [18]. Excellent inter-day reliability was documented for the hamstrings (ICC = 0.96–0.82) [19]. Excellent to good inter-day reliability was reported in lower leg muscles (ICC = 0.96–0.90 for GM, GL, Tibialis anterior muscle (TA), and Peroneus longus muscle (PL)) [20]. Baumer et al. [21] reported fair to moderate inter-day repeatability with SWE applied on the upper limb. 

**Figure 6:**
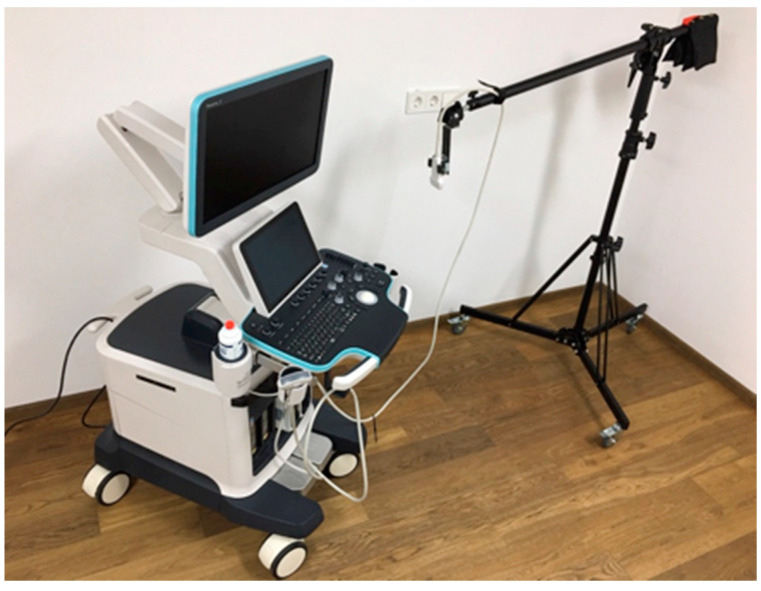
Ultrasound probe with fixation arm.

Figure 6 shows the ultrasound elastography device with holding arm for the probe. The ultrasonic probe sends a pulse (push beam) into the measuring area, and the transverse shear waves propagate at different speeds [22]. The propagation velocity Cs is given in m/s. The modulus of elasticity E is calculated using the following formula:

E = 3G = 3ρCs2. G (in kPa) is the shear modulus, which shows how the stiffness of the fabric changes. p (in kg/m^3^) indicates the density of the fabric and is assumed by most manufacturers to be 1000 kg/m^3^ [23]. 

**Figure 7:**
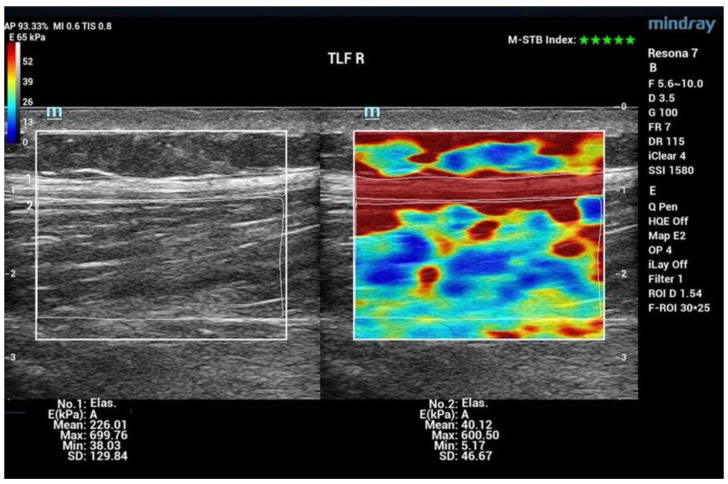
SWE Tracing and Color code. Note: Measurement region thoracolumbar fascia (TLF) and erector spinae muscle (ESp) right; (**left**): B-scan mode; (**right**): SWE; manual division of a measuring region into two measuring ranges: TLF and ESp; indication of elastic modulus (E) in kPa as Mean (average value), Max (maximum value), Min (minimum value), and SD (standard deviation). The stars at the top right of the image show the motion stability index (M-STB). The scale ranges from 0 (for poor quality) to 5 (for optimum quality).

Figure 7 shows examples of two Solographe images: Ultrasound B-scan and SWE. In order to display individual structures, such as the TLF separately from the muscle (measuring range: ESp), the measuring ranges are calculated separately by tracing.

This separation was not made for all other measurement ranges.

A joint mobility assessment was executed utilizing the Deluxe Inclinometer sourced from Performance Attainment Associates (Roseville, MI, USA). This cutting-edge device incorporates a gravitational goniometer enhanced with fluid-damped ball technology, thereby enabling meticulous and friction-free measurements. In particular, the Cervical Range of Motion Instrument (C-ROM) was enlisted to assess rotational, lateral flexion, flexion, and extension movements of the cervical spine. Simultaneously, the Back Range of Motion Instrument (B-ROM) played an important role in quantifying lateral flexion and rotation while maintaining an upright spinal posture. Moreover, the instrument facilitated the evaluation of foot flexibility (dorsiflexion) and hip joint mobility through the Leg Flexion-extension test.

During trunk flexion, the Finger-to-Floor Distance (FFD) measurement was conducted utilizing a vertical measuring board (Flex Board) affixed to a standing platform. The deepest point of the fingers was ascertained in centimeters, with the reference line (0-line) aligned with the platform’s height. Similarly, the Heel-to-Buttock Distance (HBD) was quantified in centimeters using a ruler.

The achievement of uniform pressure application was secured by integrating the MicroFet2TM Pressure Sensor, ensuring precision within a measurement accuracy range spanning from 3.6 Newtons (N) to 1320 N. For determining the distance from the heel to the buttocks, a constant force of 80 Newtons (N) was consistently applied to the shin.

The pressure pain threshold was measured with an algometer (hand force precision measuring device) from the company TesT GmbH in Newton 320.1 (N). Values were determined for two body regions (measuring range trap and TLF) and analyzed in side-by-side comparison. Measurements were performed by the same investigator at the beginning and at the end of the 12-week intervention.

Participants’ well-being was evaluated through a questionnaire, and intrapersonal comparisons were conducted using the Numeric Pain Rating Scale (NPRS). The NPRS scale (Numeric Pain Rating Scale) is a widely used method for subjective assessment of pain intensity. The measurement procedure is considered validated [24,25,26]. To streamline the analysis of pain, tension, and discomfort responses on the 0–10 NPRS scale. 35 body regions were rated in a range of 0–10 at baseline and after completion of the intervention. For statistical evaluation and intraindividual comparative analysis, the data were reduced to two statements:

There is a state of discomfort

(all statements from 1–10 were included in the evaluation).

There is an intense state of discomfort

(all statements greater than 5 were included in the evaluation).

### 2.4. Statistical Analysis Methods

The data collected from the analysis methods used (SWE, ROM, PPT, and individual feedback) were initially processed in machine-readable form using Microsoft Excel at both points in time. The subsequent statistical evaluation of the data was carried out using the open-source statistical programming language R (version 3.6.1), implemented in the editor RStudio (version 1.1.456). The statistical tests and functions described below are part of the basic R package “stats” (version 3.6.1). First, the type of statistical distribution of the measured values was examined both at T1 for the entire cohort and at T1 and T2 for the MR and CT subgroups (reference variables). The Shapiro–Wilk test, which has a high test power for sample sizes between 3 and 5000 values, was used to test for normal distribution [27]. The significance level was set at *p* = 0.05. If, within the scope of a partial evaluation, all reference values showed a normal distribution, the arithmetic mean was used for further consideration and calculations. The differences between the two mean values were determined using the *t*-test (significance level *p* = 0.05). If one or more reference variables were not normally distributed, the method of non-parametric statistics was used for further evaluation. In this case, comparisons between the reference values were based on the medians. The statistical significance of the difference between two medians was determined using the Wilcoxon–Mann–Whitney sign-rank test (significance level *p* = 0.05) [28]. To investigate possible correlations between two reference variables, the rank correlation coefficient ρ according to Spearman was used, which sensitively calculates monotonic correlations between two samples [29]. The ρ values from −1 (perfectly monotonically decreasing) to 1 (perfectly monotonically increasing) indicate the correlation. Values in the range for ρ from −0.5 to 0.5 show no or unclear correlations.

Additional parameters, which were calculated for further interpretation based on the reference values, are introduced with the corresponding slopes in the respective sections.

## 3. Results

A number of 68 participants were available for evaluation.

### 3.1. Shear Wave Elastography (SWE)

Among the 22 investigated regions, alterations in the stiffness of both muscles and fascia were observed in 17 regions as a result of the MR training. Notably, the measurement area of the trapezius muscle (Trap) exhibited the most significant change (∆E), as shown in Table 4.

It needs to be noted that Table 4 presents the percentage difference in median values between T1 and T2 specifically for the MR group. The utilization of the median as the reference value was based on the absence of normal distribution in all measurement areas. It is important to highlight that certain measurement areas did not exhibit any notable changes in stiffness.

For 13 out of 22 measurement areas, the CT training induced alterations in the stiffness of muscles and fascia, as shown in Table 5.

Table 6 comprises the difference between T1 and T2 for the CT group in median values. Similarly to the MR group, within the CT group, there were also measurement areas without notable alterations in stiffness. These specific areas with a relatively stable stiffness are omitted in the table.

The ∆E values of all 22 measurement areas of both groups were compared to evaluate stiffness changes in the myofascia.

### 3.2. Range of Motion (ROM)

For all 27 measurement areas of the body and spinal joints, ∆ROM significantly increased in the MR group.

Table 7 presents the absolute and relative changes (∆ROM) between T1 and T2 for the MR Group, encompassing diverse measurement areas. The ∆ROM in the Finger–Floor Distance measurement displays a substantial change of 5 cm (133%). Additionally, Hip Internal Rotation exhibits notable changes (∆ROM of 8°/40% for the right hip and 7°/28% for the left hip). Moreover, the Heel–Buttock Distance (FGA) shows improvements of 3 cm (20%) and 17% on both sides, whereas a consistent increase in ∆ROM in Leg Extension was measurable for both sides by 10°/16%.

A total of 16 areas out of 27 measurement areas pertaining to the body and spinal joints exhibited significant ∆ROM alterations within the CT group from T1 to T2, as illustrated in Table 8.

Changes in ∆ROM of 6° and 7° (30% and 29%) in hip internal rotation were observed for the right and the left side, respectively. Minor symmetrical changes in spinal lateral flexion (WS Latflex) and cervical spine rotation (HWS Rot) were also identified on both sides (WS Latflex 2°/5%). For the within-individual side comparison (∆ROMRL), another contrast occurs between the intervention groups (MR/CT). In the MR group, ∆ROMRL exhibited a decrease in eight out of twelve measurement areas. Conversely, three measurement areas showed an increase in ∆ROMRL. In the CT group, ∆ROMRL decreased in two out of twelve measurement areas, whereas it increased in seven measurement areas. In general, the changes in ROM within the CT group were less pronounced, in terms of both the number of measurement areas and the extent of ∆ROM, compared to the MR group.

### 3.3. Pressure Pain Threshold (PPT)

The measurement area “Trap” demonstrated a parametric distribution within the participant group, which encompassed 68 individuals. The assessment of ∆PPT did not yield any statistically significant results neither for the MR group (n = 47) nor the CT group (n = 21), Table 9. 

The measurement area “TLF” exhibits a normal distribution within the total participant group, which included 68 individuals. Significant changes were noted within the MR group (n = 47), whereas no significant changes were observed within the CT group (n = 21). Negative values mean an increase in sensitivity, and positive values accordingly indicate a decrease.

The implementation of the Mobility Routine yielded a significant enhancement in pressure sensitivity within the TLF (thoracolumbar fascia). The pressure pain threshold decreased. Changes in pressure pain thresholds were observed across all measurement ranges; however, a statistical significance was only achieved within the TLF measurement area, as illustrated in Table 10. Negative values display an increase in sensitivity and vice versa.

### 3.4. Well-Being

The sensitivity data are subjective assessments on a scale of 0–10. Each individual evaluated a comprehensive set of 35 body regions. The percentage alterations for each sensitivity aspect (∆tension, ∆pain, and ∆discomfort) were systematically examined and analyzed. Table 11 shows the changes in each sensitivity (∆tension, ∆pain, and ∆comfort) for all indications from 1–10 on the NPRS and for the indications related to the changes in severe discomfort (≥5).

**Figure 8:**
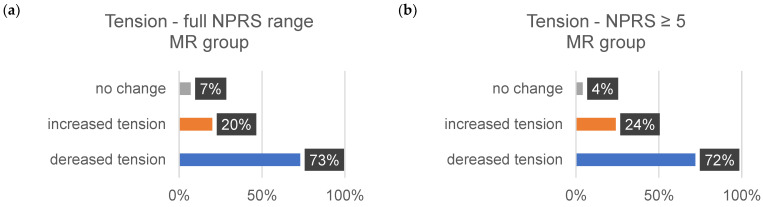
This bar chart depicts the alterations in sensitivity to tension within the MR group. Panel (**a**) offers a comprehensive view of all ratings ranging from 0 to 10 on the NPRS (Numeric Pain Rating Scale). In contrast, panel (**b**) shows values of ≥5 on the NPRS.

**Figure 9:**
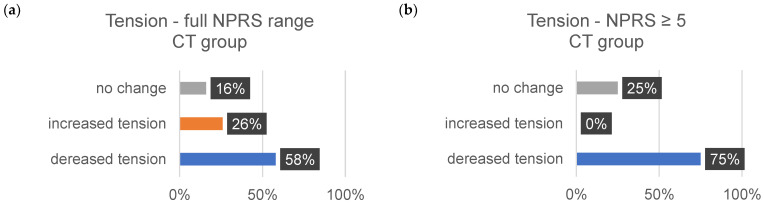
The provided bar chart illustrates the changes in sensitivity to tension within the CT group. Panel (**a**) provides a holistic representation of all ratings spanning from 0 to 10 on the NPRS. Conversely, panel (**b**) exclusively displays values ≥ 5 on the NPRS.

**Figure 10:**
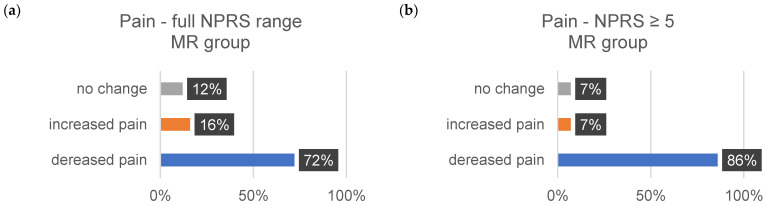
The provided bar chart illustrates the changes in sensitivity to pain within the MR group. Panel (**a**) provides a holistic representation of all ratings spanning from 0 to 10 on the NPRS. Conversely, panel (**b**) exclusively displays values ≥ 5 on the NPRS.

**Figure 11:**
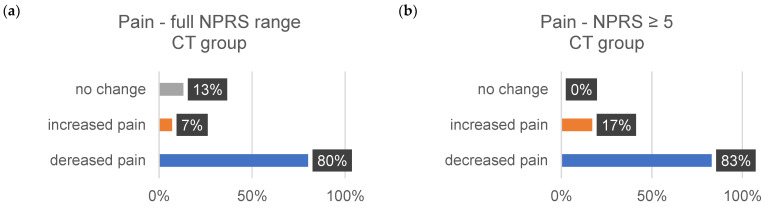
The provided bar chart illustrates the changes in sensitivity to pain within the CT group. Panel (**a**) provides a holistic representation of all ratings spanning from 0 to 10 on the NPRS. Conversely, panel (**b**) exclusively displays values ≥ 5 on the NPRS.

**Figure 12:**
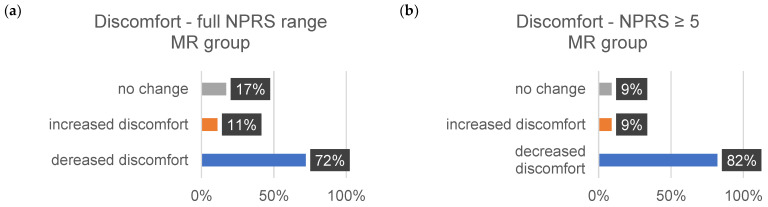
The provided bar chart illustrates the changes in sensitivity to discomfort within the MR group. Panel (**a**) provides a holistic representation of all ratings spanning from 0 to 10 on the NPRS. Conversely, panel (**b**) exclusively displays values ≥ 5 on the NPRS.

**Figure 13:**
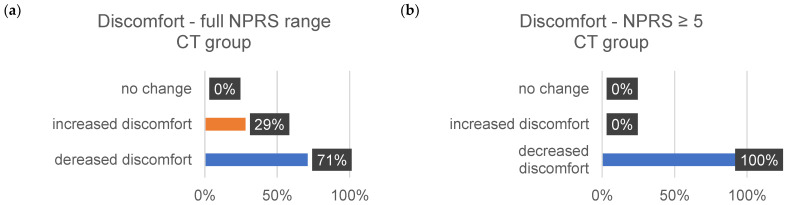
The provided bar chart illustrates the changes in sensitivity to discomfort within the CT group. Panel (**a**) provides a holistic representation of all ratings spanning from 0 to 10 on the NPRS. Conversely, panel (**b**) exclusively displays values ≥ 5 on the NPRS.

The subsequent bar charts in Figure 8, Figure 9, Figure 10, Figure 11, Figure 12 and Figure 13 visually represent the percentage changes (∆) for all sensitivities.

In summary, according to the stated perception, both interventions led to improved well-being. In the MR group, all values for the increase in well-being were above 72%. Of particular note were the high percentage values for the decrease in individual intense pain perception and discomfort (86%/82%) in the MR group. In the CT group, the perception of general overarching tension decreased proportionally by only 58%. The data for the reduction in intense discomfort could not be included in the evaluation because only one subject provided information. A correlation with the results of the other measurement methods could not be established.

## 4. Discussion

The objective of this study was to assess the impact of a diverse mobility training program (referred to as Mobility Routine—MR) on alterations in tissue stiffness, mobility, and subjective well-being. In the following sections of this paper, the respective results will be discussed in detail and related to the different loading standards.

### 4.1. Elastic Modulus Analysis

The assessment of the Myofascial Elastic Modulus (E) revealed a notable increase in myofascial stiffness in both intervention groups. The increase in E in the CT group was more pronounced for fewer measurement ranges. This is possibly due to a more consistent and cyclic loading by repetitive movements patterns and thus fewer body regions being exposed to higher stress levels in CT training. In the MR group, E was moderately increased for a greater number of measurement areas, indicating a consistent response across sides.

The current database for the analysis of myofascia using SWE is poor. Accordingly, there is a lack of reference values of E (in kPa) that would allow for an evaluation of the results. The greater increase in E for fewer measurement ranges in the CT group suggests that repetitive movements of the same kind result in greater tissue stiffness. More studies and development of comprehensive data sets would allow for assessments of the absolute value of E. For the current study, the results were analyzed in the intraindividual comparison of T1 to T2 and in the side-to-side comparison (right-left/RL). The data collected are used for basic research and are considered to contribute to a better assessment and evaluation of the absolute value of E in the future.

### 4.2. Range of Motion (ROM)

The MR training led to improvements in joint mobility across body and spinal joints and was superior to CT training concerning Range of Motion (ROM) and the number of affected measurement areas (MR: 27/27; CT: 16/27). The alignment of intra-individual right-left disparities further solidified the superiority of MR training. In eight out of twelve (only the bilateral measuring ranges were considered) measurement areas, a reduction in intra-individual right-left differences was observed. MR training proved to be significantly more effective than CT training in compensating for imbalances. For instance, MR training contributed to balancing side-to-side differences in the hip, whereas CT training accentuated the asymmetry between body sides.

In this study, no significant correlation was detected between Shear Wave Elastography (SWE) and ROM results.

### 4.3. Pressure Pain Threshold (PPT)

The measurement of the pressure pain threshold (PPT) was conducted on two specific body regions, specifically the lower back (TLF) and the upper edge of the trapezius muscle (Trap). The results in the trap measurement area were rather inconsistent, as it was difficult to determine by the subject, and these were thereby heterogeneous and not significant.

### 4.4. Well-Being

The analysis of the questionnaire yielded significant reductions in the perception of tension, pain, and discomfort. In the MR group, discomfort decreased by more than 76 ± 6%, whereas a similar reduction of 73 ± 11% was observed in the CT group. Only small increases in discomfort (MR: 15 ± 9%; CT: 16 ± 10%) were observed.

Both interventions led to a significant enhancement in individual well-being, with MR training showing a slightly more pronounced effectiveness than CT training. Multiple studies [30,31,32,33] affirm the relationship between exercise and pain reduction. The key lies in selecting a kind of movement with moderate intensity, as intense exertion can contribute to the accumulation of pro-inflammatory cytokines associated with the development of disease and pain [34,35,36,37].

## 5. Conclusions

In summary, our study demonstrates significant advancements in mobility, tissue stiffness, and subjective well-being following a 12-week Mobility Routine (MR) intervention. The MR proved superior to conventional training (CT) in enhancing mobility, as evidenced by a significant increase in Range of Motion (ROM) across all 27 measurement areas. The observed harmonizing effect of MR on imbalances in intra-individual right-left comparisons further supports its efficacy. Results from Shear Wave Elastography (SWE) indicated a moderate and homogeneous increase in the elastic modulus (E) in the MR group, contrasting with stiffness changes induced by CT. While the optimal tension and elasticity for various body regions remain unclear, our extensive dataset significantly contributes to future standardization efforts.

Of note is the examination of the Pressure Pain Threshold (PPT) in the thoracolumbar fascia (TLF) for the MR group, revealing increased sensitivity, possibly associated with the impact of MR on mechanoreceptors and nociceptors. Future investigations should explore PPT in multiple regions to enhance statistical robustness.

The observed enhancement in overall well-being and consistent reduction in tension, pain, and discomfort by more than 70% underscore the holistic benefits of MR. Our results provide compelling evidence for the positive impact of MR on myofascial structures.

In a broader context, this study advocates for a more comprehensive understanding of fascial physiology and emphasizes the need for ongoing research on tissue overload and inflammation. The intervention study supports the potential of mobility training to alleviate the adverse effects of intense physical demands, paving the way for further exploration in preventive and rehabilitative contexts. Future studies should consider diverse cohorts, including individuals with chronic back pain, to validate and expand the implications of the Mobility Routine. This research contributes to the growing knowledge in sports science, providing insights for optimizing training approaches for enhanced health, performance, and injury prevention.

## Figures and Tables

**Table 1:** Anthropometrical data for the cohort from the State Criminal Investigation Office/Special Operations Command (SEK).

Parameter	Intervention Group I (MR)	Intervention Group II (CT)
Number of Participants (n)	47	21
Gender (Male)	47	21
Age (years)	28.4 ± 4.6	29.1 ± 3.8
Weight (kg)	79.6 ± 9.3	81.2 ± 10.5
Height (cm)	181.5 ± 6.3	179.8 ± 5.9
Duration of Intervention (weeks)	12	12
Physical Workload (hours/week)	24.7 ± 2.9	24.5 ± 3.1
Weight of Protective Gear (kg)	33.5 ± 2.1	33.4 ± 2.3

Note: Data presented as mean ± standard deviation.

**Table 2:** Weight of protective clothing.

Protective Clothing	Weight
Total weight during training	27.6 kg
Total weight in use	33.5 kg
Incl. paramedic rucksack	45.6 kg

**Table 3:** Measuring ranges—SWE (the measurement positions were determined individually reproducibly on the basis of anatomical features using centimeter measures).

	Region	Description	Subject- Position
1	M. trapezius Trap. L	A total of 10 cm paravertebral from the center of the cervical spine acromion	sitting
2	M. trapezius Trap R	A total of 10 cm paravertebral from the center of the cervical spine acromion	sitting
3	Plantar fascia PLF L	Calcaneus	prone position
4	Plantar fascia PLF R	Calcaneus	prone position
5	Thoracolumbar fascia TLF L4/5 L	Line ilium upper margin A total of 2 cm paravertebral proc. spinous process	prone position
6	Thoracolumbar fascia TLF L4/5 R	Line ilium upper margin A total of 2 cm paravertebral spinous process	prone position
7	M. gluteus medius GlutMed L	A total of 2.5 cm distal to the center of the crista iliac	prone position
8	M. gluteus medius GlutMed R	A total of 2.5 cm distal to the center of the crista iliac	prone position
9	M. gluteus maximus/GlutMax L	Midpoint between greater trochanter and sacrum	prone position
10	M. gluteus maximus/GlutMax R	Midpoint between greater trochanter and sacrum	prone position
11	M. biceps femoris BicFem L	A total of 20 cm from the fibula head in the direction Tuber ischiadicum	prone position
12	M. biceps femoris BicFem R	A total of 20 cm from the fibula head in the direction Tuber ischiadicum	prone position
13	M. gastrocnemius lateralis/Gastroc L	A total of 12 cm below the popliteal fossa, lateral calf	prone position
14	M. gastrocnemius lateralis/Gastroc R	A total of 12 cm below the popliteal fossa, lateral calf	prone position
15	Mm. adductor ADD L	A total of 20 cm from adductor tuberculum dist. femur towards the pubic tubercle	supine position
16	Mm. adductor ADD R	20 cm from adductor tuberculum dist. femur towards pubic tubercle	supine position
17	M. rectus femoris RFM L	0 cm from the upper edge of the patella in direction. Spina iliaca anterior superior (SIAS)	supine position
18	M. rectus femoris RFM R	0 cm from the upper edge of the patella in direction. Spina iliaca anterior superior (SIAS)	supine position
19	M. tibialis anterior TibA L	9 cm below the patella, 1 finger width lateral to the margo anterior of the tibia	supine position
20	M. tibialis anterior TibA L	9 cm below the patella, 1 finger width lateral to the margo anterior of the tibia	supine position

**Table 4:** Changes in Elastic Modulus (T1 to T2)—MR Group (the table displays the measurement areas (L = left, R = right) in the MR group T1/T2 (n = 47), where a significant change in the elasticity modulus ∆E was observed. A “+” before E indicates an increase, whereas a “–“ indicates a decrease. The significance level (*p*-value) is 0.05).

Measurement Area	∆E (%)	*p*-Value
M. trapezius		
Trap Left	+48	<0.001
Trap Right	+61	<0.001
Mm. Adductor		
ADD Left	+36	<0.001
ADD Right	+29	<0.001
M. biceps femoris		
BicFem Left	+19	<0.001
BicFem Right	+43	<0.001
M. gluteus medius		
GlMed Left	+25	<0.001
GlMed Right	+13	0.007
M. gastrocnemius lateralis		
GaLat Right	+18	<0.001
Thoracolumbar fascia		
TLF Left	+15	0.039
TLF Right	+17	0.003
M. erector spinae		
ESp Left	+15	0.001
ESp Right	+14	<0.001
M. biceps femoris		
RFM Left	+7	0.012
RFM Right	+17	<0.001
M. gluteus maximus		
GlMax Right	+10	0.006
M. anterior tibialis		
TibA Left	−9	0.014

**Table 5:** Changes in Elastic Modulus (T1 to T2)—CT Group.

Measurement Area	∆E (%)	*p*-Value
M. trapezius		
Trap Left	+94	<0.001
Trap Right	+77	<0.001
Thoracolumbar fascia		
TLF Left	+44	<0.001
M. biceps femoris		
RFM Right	+35	0.001
Mm. adductor		
ADD Left	+31	<0.001
ADD Right	+40	0.002
M. gastrocnemius lateralis		
GaLat Right	+31	0.007
GaLat Right	+31	0.007
M. biceps femoris		
BicFem Left	+31	0.002
BicFem Right	+31	<0.001
M. gluteus maximus		
GMax Right	+19	0.004
M. erector spinae		
ESp Left	+0.9	0.010
M. gluteus medius		
GlMed Left	+0.6	0.019

**Table 6:** Change in Elastic Modulus (T1 to T2)—MR-CT Comparison (the table shows the comparison of ∆E for the MR and CT groups. The * indicates that the difference value is based on a parametric distribution of the baseline data).

Measurement Area	∆E (%) MR	∆E (%) CT	MR > CT CT > MR
M. Biceps femoris			
BicFem R	+43	+40	MR > CT
Mm. adductor muscle			
ADD R	+29	+18	MR > CT
M. gluteus medius muscle			
GlMed L	+25	+6	MR > CT
Thoracolumbar fascia			
TLF R	+17	+10 *	MR > CT
M. erector spinae			
ESp L	+15	+9	MR > CT
ESp R	+14	+5 *	MR > CT
M. gluteus medius			
GlMed R	+13	0 *	MR > CT
M. rectus femoris			
RFM L	+7	0 *	MR > CT
M. trapezius			
Trap L	+48	+94	CT > MR
Thoracolumbar fascia			
TLF L	+15	+44	CT > MR
M. gastrocnemius lateralis			
GaLat L	+9 *	+31	CT > MR
M. rectus femoris			
RFM R	+17	+35	CT > MR
M. trapezius			
Trap R	+61	+77 *	CT > MR
Mm. adductor			
ADD L	+36	+50	CT > MR
M. gastrocnemius lateralis			
GaLat R	+18	+31	CT > MR
M. anterior tibialis			
TibA L	−9	+5 *	CT > MR
M. biceps femoris			
BicFem L	+19	+31	CT > MR
M. gluteus maximus			
GlMax R	+10	+19	CT > MR
M. anterior tibialis			
TibA L	−9	+5 *	CT > MR
M. gluteus maximus			
GlMax L	−7	0 *	CT > MR
M. anterior tibialis			
TibA R	+5 *	+7 *	CT > MR
Plantar fascia			
PLF L	0 *	1 *	CT > MR
Plantar fascia			
PLF R	−9 *	−8 *	CT > MR

Note: For eight measurement areas (Rows 1–8), the MR group exhibited a larger ∆E than the CT group. Conversely, for 14 measurement areas (Rows 9–22), the ∆E in the CT group was higher. CT training appears to increase stiffness to a greater degree.

**Table 7:** Change in ROM T1 T2—MR Group (Table 7 displays the Range of Motion (∆ROM) for the MR group, consisting of 47 participants. The absolute results are presented in degrees or centimeters (* indicated in centimeters), whereas the relative results are expressed in percentages. A total of 27 measurement areas were analyzed, of which thirteen were bilateral in nature (significance level = 0.05)).

Measurement Area	MT1	MT2	∆ROM Absolute (°/cm)	∆ROM Relative (%)	*p*-Value (<0.05)
Finger–Floor Distance					
FBA	4 *	9 *	5 *	133	0.000
Hip Internal Rotation					
Hip IR R	20	28	8	40	0.000
Foot Dorsiflexion 0°					
Do 0° R	13	18	5	38	0.001
Foot Dorsiflexion 90°					
Do 90° R	22	30	8	36	0.001
Hip Internal Rotation					
Hip IR L	25	32	7	28	0.000
Spinal Rotation					
WS Rot L	30	38	8	27	0.028
Foot Dorsiflexion 0°					
Do 0° L	12	15	3	25	0.000
Heel–Glute Distance					
FGA R	15	12	3 *	20	0.000
Heel–Glute Distance					
FGA L	14.5	12	3 *	17	0.000
Leg Extension					
LegExt L	62	72	10	16	0.000
LegExt R	62	72	10	16	0.000
Shoulder Internal Rotation					
Shoulder IR L	55	62	7	13	0.000
Shoulder External Rotation					
Shoulder AR R	76	85	9	12	0.000
Shoulder Internal Rotation					
Shoulder IR R	59	65	6	10	0.001
Shoulder External Rotation					
Shoulder AR L	82	89	7	9	0.001
Hip External Rotation					
Hip AR L	39	42	3	8	0.008
Spinal Lateral Flexion					
WS Latflex L	39	42	3	8	0.000
Spinal Rotation					
WS Rot R	30	32	2	7	0.024
Cervical Spine Flexion					
C-spine Flexion	59	63	4	7	0.000
C-spine Lateral Flexion					
C-spine Latflex R	40	42	2	5	0.000
Cervical Spine Lateral Flexion					
C-spine Latflex L	42	44	2	5	0.003
Cervical Spine Rotation					
C-spine Rot R	70	73	3	4	0.000
Hip External Rotation					
Hip ext. rot. R	43	44	1	2	0.000
Cervical Spine Extension					
C-spine Extension	78	79	1	1	0.028
Cervical Spine Rotation					
C-spine Rot L	79	78	0	0	0.031
Cervical Spine Lateral Flexion					
C-spine Latflex R	45	45	0	0	0.000
Foot Dorsiflexion 90°					
Foot d-flex 90° L	20	20	0	0	0.003

**Table 8:** Change in ROM T1 T2—CT group (Table 8 illustrates the Range of Motion (∆ROM) for the CT group, comprising 21 participants. The results are presented in degrees or centimeters (* indicated in centimeters), whereas relative results are expressed in percentages. A total of 27 measurement areas were analyzed, with thirteen of them being bilateral (significance level = 0.05)).

Measurement Area	MT1	MT2	∆ROM Absolute (°/cm)	∆ROM Relative (%)	*p*-Value (<0.05)
Finger–Bottom Distance					
FBA	3.6	7	3 *	94	0.000
Hip Internal Rotation					
Hip IR R	20	26	6	30	0.050
Hip IR L	24	31	7	29	0.000
Heel–Buttock Distance					
FGA R	16	13	3 *	19	0.000
Shoulder Internal Rotation					
Shoulder IR L	53	61	8	15	0.001
Heel–Buttock Distance					
FGA L	16.5	15	2 *	12	0.002
Shoulder External Rotation					
Shoulder ext. rot L	81	88	7	9	0.004
Shoulder int. rot. R	57	62	5	9	0.014
Cervical Spine Flexion					
C-spine Latflex	59	64	5	8	0.021
Foot Dorsiflexion 90°					
Foot do-fl 90° L	17	18	1	6	0.018
Shoulder External Rotation					
Shoulder ext. rot. R	79	84	5	6	0.036
Spinal Lateral Flexion					
Spine Latflex L	40	42	2	5	0.001
Spine Latflex R	39	41	2	5	0.001
Cervical Spine Rotation					
C-spine Rot L	70	73	3	4	0.005
C-spine Rot R	70	72	2	3	0.029
Foot Dorsiflexion 0°					
Foot do-fl 0° L	11	11	0	0	0.019

**Table 9:** Change in pressure pain threshold T1 T2—Trapezius muscle.

Measurement Area	∆PPT (N) T1–T2 MR	*p*-Value (<0.05)	∆PPT (N) T1–T2 CT	*p*-Value (<0.05)	∆PPT (N) T1–T2 MR-CT
Trapezius Muscle					
Trap L	−3	0.420	+2	0.530	−MR +CT
Trap R	+1	0.696	−4	0.726	+MR −CT

**Table 10:** Change in pressure pain threshold T1 T2—thoracolumbar fascia.

Measurement Area	∆PPT (N) T1–T2 MR	*p*-Value (<0.05)	∆PPT (N) T1–T2 CT	*p*-Value (<0.05)	∆PPT (N) T1–T2 MR-CT
Thoracolumbar fascia					
TLF L	−22.4	0.002	−3.4	0.741	−MR −CT
TLF R	−24	0.001	-12	0.308	−MR −CT

**Table 11:** Changes in sensitivity T1 T2—MR-CT.

	∆Tension (%)		∆Pain (%)		∆Discomfort (%)	
	1–10	≥5	1–10	≥5	1–10	≥5
decrease						
MR	73	72	72	86	72	82
CT	58	75	80	83	71	100
increase						
MR	20	24	16	7	11	29
CT	26	0	7	17	9	0
no change						
MR	7	4	12	7	17	9
CT	16	25	13	0	9	0

## Data Availability

The research paper, including all data sets, is available for public inspection in the library of the Foundation University of Hildesheim (https://doi.org/10.25528/099).
